# Expression of NRG1 and its receptors in human bladder cancer

**DOI:** 10.1038/bjc.2011.39

**Published:** 2011-03-01

**Authors:** J A Forster, A B Paul, P Harnden, M A Knowles

**Affiliations:** 1 Cancer Research UK Clinical Centre, St James University Hospital, Beckett Street, Leeds LS9 7TF, UK; 2 Pyrah Department of Urology, St James University Hospital, Beckett Street, Leeds LS9 7TF, UK

**Keywords:** neuregulin, bladder cancer, EGFR, ERBB2, ERBB3

## Abstract

**Background:**

Therapies targeting ERBB2 have shown success in the clinic. However, response is not determined solely by expression of ERBB2. Levels of ERBB3, its preferred heterodimerisation partner and ERBB ligands may also have a role.

**Methods:**

We measured NRG1 expression by real-time quantitative RT–PCR and ERBB receptors by western blotting and immunohistochemistry in bladder tumours and cell lines.

**Results:**

NRG1*α* and NRG1*β* showed significant coordinate expression. NRG1*β* was upregulated in 78% of cell lines. In tumours, there was a greater range of expression with a trend towards increased NRG1*α* with higher stage and grade. Increased expression of ERBB proteins was detected in 15% (EGFR), 20% (ERBB2), 41% (ERBB3) and 0% (ERBB4) of cell lines. High EGFR expression was detected in 28% of tumours, associated with grade and stage (*P*=0.05; *P*=0.04). Moderate or high expression of ERBB2 was detected in 22% and was associated with stage (*P*=0.025). Cytoplasmic ERBB3 was associated with high tumour grade (*P*=0.01) and with ERBB2 positivity. In cell lines, NRG1*β* expression was significantly inversely related to ERBB3, but this was not confirmed in tumours.

**Conclusion:**

There is a wide spectrum of NRG1 and ERBB receptor expression in bladder cancer. In advanced tumours, EGFR, ERBB2 and ERBB3 upregulation is common and there is a relationship between expression of ERBB2 and ERBB3 but not the NRG1 ligand.

Bladder cancer is the fourth most common malignancy in males and the ninth most common in females in the United Kingdom, with 10 090 new cases in 2007 (http://info.cancerresearchuk.org/cancerstats/types/bladder/incidence/). At presentation, 70–80% of patients have non-muscle-invasive urothelial carcinoma (UC; stage Ta or T1) and 20–30% have invasive disease (⩾stage T2). For the latter, 5-year survival rate is ∼50% and, although current chemotherapy and radiotherapy regimes are effective in some patients, response rates have plateaued and novel therapeutic approaches are urgently needed.

Overexpression of the ERBB receptor tyrosine kinases in human cancer has led to the development of targeted therapies (Hynes and Lane, 2005). ERBB2 is overexpressed in 20–30% of breast cancers, and the monoclonal antibody Trastuzumab (Herceptin) that targets ERBB2 has been used with success to treat these (Slamon *et al*, 2001). As some bladder tumours overexpress ERBB2 (Chow *et al*, 2001), it has been suggested that these may respond to ERBB2 inhibitors (Latif *et al*, 2004) and therefore clinical studies have been initiated (Small *et al*, 2003). A study of Trastuzumab, paclitaxel, carboplatin and gemcitabine in advanced ERBB2-positive UC has been reported, with an overall response rate of 70% (Hussain *et al*, 2007). However, a randomised trial is required to assess the contribution of Trastuzumab to such responses. A phase I/II study of paclitaxel and radiotherapy, with or without Trastuzumab, is now underway in patients who have undergone previous resection for muscle-invasive UC (NCT00005831).

Not all breast cancers that overexpress ERBB2 respond to Trastuzumab (Vogel and Franco, 2003). In some cases, lack of response is related to downstream activation of the PI3K pathway through mechanisms such as PTEN inactivation (Nagata *et al*, 2004). ERBB3 is known to couple ERBB2 to the PI3K pathway (Junttila *et al*, 2009) and the crucial role of ERBB3 in mediating the effects of activated ERBB2 is now clear (Lee-Hoeflich *et al*, 2008; Baselga and Swain, 2009). Altered expression of the ligand neuregulin may also determine sensitivity to ERBB2 inhibitors and identify a novel group of patients who could benefit from ERBB-targeted therapies (Yuste *et al*, 2005; Menendez *et al*, 2006).

Neuregulin 1 (*NRG1*) is a member of the epidermal growth factor (EGF) family (Yarden, 2001). At least 10 *NRG1* isoforms have been identified (Stove and Bracke, 2004) (Supplementary Figure 1). Types I, II and III have distinct amino termini that contain an Ig-like domain in types I and II (Li and Loeb, 2001). The N terminus of type III isoforms contains a cysteine-rich domain with a transmembrane domain. All isoforms contain an EGF-like domain, which is essential (and sufficient) for receptor activation (Alroy and Yarden, 1997). Alternative splicing in this domain gives rise to *α-* and *β*-variants. Alternative splicing in the extracellular juxtamembrane region gives rise to four further variants. Finally, alternative splicing in the cytoplasmic tail (C-terminal) leads to a, b or c variants.

NRG1 binds ERBB3 and ERBB4 and NRG1*β* binds ERBB3 with a higher affinity than NRG1*α* (Jones *et al*, 1999). As there is no ligand for ERBB2, heterodimerisation with a ligand-bound receptor is required for signalling (Klapper *et al*, 1999), and ERBB2 is the preferred binding partner for other ligand-bound ERBB receptors (Garrett *et al*, 2003; Franklin *et al*, 2004). As ERBB3 is devoid of intrinsic kinase activity, it requires dimerisation with another ERBB receptor for phosphorylation (Guy *et al*, 1994) and ERBB2–ERBB3 heterodimers provide the most mitogenic and angiogenic signals (Alimandi *et al*, 1995).

Increased expression of NRG1 proteins has been reported in several cancers. However, there is little data on expression in normal urothelium or bladder tumours and reports are inconsistent. De Boer *et al* (1997) showed that expression of different NRG1 isoforms varied in different cell layers of the normal and malignant bladder. In the normal urothelium, expression of NRG1*α* and NRG1*β*1 isoforms was highest in the differentiated superficial cells and NRG1*α* and NRG1*β* mRNA was detected in 90 and 61% of tumours, respectively, with a negative trend between NRG1*β* expression and survival (Memon *et al*, 2004). In contrast, Amsellem-Ouazana *et al* (2006) did not detect NRG1 in 5 normal bladder and 73 tumour samples.

*NRG1* maps to chromosome arm 8p, a region of common genomic alteration in human tumours including bladder. Loss of heterozygosity (LOH) of 8p is found in muscle-invasive UC (Wagner *et al*, 1997; Ohgaki *et al*, 1999; Choi *et al*, 2000), and in bladder and other tumour types 8p LOH is associated with worse prognosis. In breast cancer, several breakpoints have been identified close to or within *NRG1* (Huang *et al*, 2004) and recent identification of promoter hypermethylation suggests a possible suppressor role (Chua *et al*, 2009). However, upregulation has also been reported (McIntyre *et al*, 2009).

The reported expression of the ERBB family of receptors in normal and malignant bladder also differs markedly between studies, and these are difficult to compare because of differences in methods, antibodies and scoring criteria. Before ERBB-targeted therapies can be rationally applied in bladder cancer, clear data on the expression of the different receptors and relevant ligands are required. The objectives of this study were to determine expression of the two major isoforms of NRG1 and the ERBB family of receptors in human bladder cancer cell lines and tumour tissues. We have applied the criteria used for scoring of ERBB2 immunohistochemistry in breast cancer to obtain standardised assessment of expression and hence a more rational prediction of the proportion of bladder cancer patients who may benefit from ERBB2-targeted therapy.

## Materials and methods

### Patient recruitment and tissue collection

Ethical approval was obtained from the Leeds (East) Local Research and Ethics Committee, and informed consent for tissue donation was obtained from patients at St James's University Hospital, Leeds. Tumour tissues obtained by cold-cup biopsy at cystoscopy or from cystectomy were snap-frozen. Fifty-nine fresh samples were used (23 pTaG2; 4 pTaG3; 5 pT1G2; 14 pT1G3; 10 pT2G3; and 3 T4G3).

### Cell lines

Primary normal human urothelial cells (NHUC) and telomerase immortalised NHUC (TERT-NHUC) were maintained as described (Chapman *et al*, 2006). Thirty-four UC cell lines (Supplementary Table 1) were grown in standard media at 37°C in 5% CO_2_.

### mRNA extraction and cDNA preparation

RNA was extracted from cell lines using the RNeasy mini kit (Qiagen, Crawley, UK), and from tissue sections containing >80% tumour cells using the Picopure RNA isolation kit (Arcturus Bioscience, Mountain View, CA, USA). For cDNA synthesis, the Clontech Advantage 1st strand kit (Clontech, Palo Alto, CA, USA) was used.

### Quantitative real-time RT–PCR

Quantitative RT–PCR (qRT–PCR) used SYBR Green I as reporter and ROX as reference dye (Perkin-Elmer, Applied Biosystems, Cheshire, UK). Fluorescence was detected by an ABI prism 7700 Sequence Detection System (Applied Biosystems) and analysed using the Sequence Detector System 1.9 software (Applied Biosystems). Succinate dehydrogenase (*SDHA*) and hypoxanthine phosphoribosyl-transferase1 (*HPRT1*) were used as controls. Primers were designed using Primer Express software (Applied Biosystems; Supplementary Table 2). Preliminary experiments over a range of concentrations of reverse transcribed Universal reference RNA (Stratagene, La Jolla, USA) were conducted to determine the efficiency of the PCR reaction for each set of primers and the comparative C_T_ (ΔΔC_T_) method was used for analysis (Livak and Schmittgen, 2001). The cDNA from pooled pure populations of uncultured normal human urothelial cells was used as a control for cell line analysis.

### Western blotting

Cells were lysed in ESB buffer (0.12M Tris-HCL, pH 6.8; 20% glycerol; 4% SDS) and protein concentration measured using the Pierce Protein Assay (Rockford, IL, USA). Antibodies used were anti-EGFR (1005 sc-03 Santa Cruz, Santa Cruz, CA, USA), ERBB2 (A0485 Dako Cytomation, Ely, UK), ERBB3 (c-17 sc-285 Santa Cruz) and ERBB4 (RB-284 Labvision, Fremont, CA, USA). Anti-Ku-70 (Santa Cruz) was used as a loading control. For quantification, films were scanned on a Personal Densitometer SI (Molecular dynamics/Amersham Biosciences, Little Chalfont, UK) and band densities were calculated with ImageQuant software (Molecular dynamics/Amersham Biosciences).

### Immunohistochemistry

Fifty-four paraffin-embedded tumour tissues were studied. Three normal ureters and cell line pellets known to be positive or negative for each antigen were used as controls. For ERBB2, a strongly positive breast cancer tissue was used as a control in each run.

Endogenous peroxidase activity was blocked in 3% hydrogen peroxide, sections were boiled in antigen unmasking solution (Vector Laboratories, Peterborough, UK) for 2 min (EGFR and ERBB2) and blocked with either 10% (v/v) casein (ERBB2) or avidin/biotin solutions (Vector Laboratories) (other antigens), followed by treatment with 10% normal goat serum (Dako Cytomation). Primary antibodies for EGFR (NCL-EGFR-384, Novocastra, Newcastle, UK), ERBB2 (A0485, Dako Cytomation), ERBB3 (RTJ2 (Rajkumar *et al*, 1995); supplied by Cancer Research UK) and ERBB4 (c-18 sc-283 Santa Cruz) were applied for 60 min. Following incubation with Envision (Dako Cytomation) (ERBB2) or secondary anti-species antibody, followed by treatment with Streptavidin–biotin enzyme complex (StreptAB-Complex, Dako Cytomation), binding was visualised using 3,3′diamino-benzidine tetrahydrochloride (Vector Laboratories).

Scoring was performed by JF, PH and MK. In the absence of full concordance, slides were reviewed and a decision taken. Membrane staining was considered as positive for EGFR and ERBB2. For ERBB3, membrane staining was not observed and cytoplasmic and nuclear reactivity were scored separately. Scoring criteria for EGFR and ERBB2 were as used for ERBB2 in breast cancer tissues and a multitissue breast reference section was used to guide scoring (0, no membrane staining; 1+, weak membrane staining only; 2+, strong membrane staining in some cells; 3+, strong membrane staining in all cells). For ERBB3, initial scoring for cytoplasmic and nuclear staining was according to both intensity and frequency (weak, moderate, strong; <5%, 5–50%, >50% of cells). This was subsequently refined for cytoplasmic staining to score as positive tumours in which >5% of cells showed moderate or strong staining, and for nuclear staining to score those in which >50% of cells showed moderate or strong staining.

### Statistical analysis

The statistical software Stata (v10, StataCorp, College Station, TX, USA) was used for data handling and analyses. The relationship between NRG1*α* and NRG1*β* expression and between NRG1*α*/*β* and EGFR/ERBB2/ERBB3 expression was assessed using Spearman's rank correlation. The Mann–Whitney test was used to assess the relationships between NRG1*α*/*β* expression and tumour stage (Ta compared with ⩾T1 tumours) and grade (grade 2 compared with grade 3). Fisher's exact test was used to assess the relationship between tumour grade and stage and immunohistochemistry score.

## Results

### NRG1 expression in normal urothelium, UC cell lines and tumours

Quantitative RT–PCR on uncultured urothelial cells isolated from five normal healthy ureters and a pooled sample composed of equal amounts of these samples showed no significant differences in expression of NRG1*α* and NRG1*β* (Supplementary Figure 2). In all samples, levels of both isoforms were lower than levels of the control genes.

**Figure 1:**
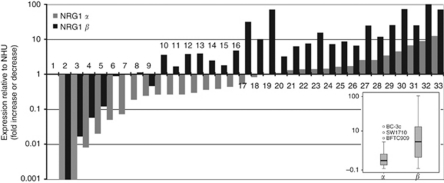
Real-time RT–PCR measurements of NRG1*α* and NRG1*β* levels in bladder cell lines. Results are expressed relative to a pooled normal urothelial cell RNA sample (1). Cell lines analysed: 2, VMCUBIII; 3, JO’N; 4, HT1376; 5, HT1197; 6, DSH1; 7, 647V; 8, RT4; 9, 97-29; 10, 97-24; 11, 94-10; 12, KU19-19; 13, JMSU1; 14, RT112; 15, 96-1; 16, SD; 17, CAL29; 18, 97-6; 19, SCaBER; 20, 97-18; 21, 97-7; 22, T24; 23, 5637; 24, 97-1; 25, VMCUBII; 26, J82; 27, UMUC3; 28, 253J; 29, BFTC905; 30, TCCSUP; 31, BFTC909; 32, SW1710; 33, BC3C. Inset shows the range of expression of NRG1*α* and *β* as a boxplot. Horizontal line indicates median value.

NRG1α and NRG1*β* expression in 32 UC cell lines relative to cultured NHUC controls is shown in Figure 1. There was significant coordinate expression of the two isoforms (Spearman's rank correlation 0.889; *P*<0.001). Overall, NRG1*β* showed higher expression than NRG1*α* and was upregulated more frequently. Twenty-five of 32 cell lines showed NRG1*β* levels ⩾1.5 higher than controls, and in several cases the levels were 50- to 100-fold higher.

**Figure 2:**
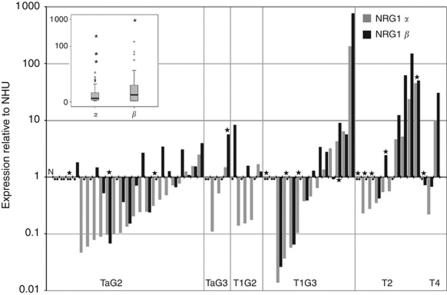
Real-time RT–PCR measurements of NRG1*α* and NRG1*β* levels in tumour samples. Results are expressed relative to a pooled normal urothelial cell RNA sample (N). Samples are grouped according to tumour grade and stage. 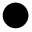, no expression detected; *, samples that showed high levels of expression of ERBB2. Inset shows the range of expression of NRG1*α* and *β* as a boxplot. Horizontal line indicates median value.

Quantitative RT–PCR on cDNA from 59 tumours revealed more varied expression levels than in cell lines, ranging from undetectable in some tumours to 770-fold higher expression of NRG1*β* in one tumour compared with normal urothelium (Figure 2). Although NRG1*β* was more highly expressed than NRG1*α*, this was not as significant as in cell lines. As in cell lines, there was a statistically significant correlation between expression of NRG1*α* and NRG1*β* (Spearman's rank correlation, *r*=0.680, *P*<0.0001). There was a tendency for NRG1*α* upregulation in high-grade and stage tumours, although this did not reach significance (for grade *P*=0.08, Mann–Whitney test; for stage *P*=0.06, Spearman's rank correlation test). There was no significant relationship between NRG1*β* and grade or stage (for grade *P*=0.44, Mann–Whitney test; for stage *P*=0.13, Spearman's rank correlation test).

We have detailed information on 8p genomic alterations in all of the cell lines studied (Williams *et al*, 2010). Expression of NRG1 bore no obvious relationship to either copy number in the region of the gene or to breakpoints in the region.

### ERBB protein expression

**Figure 3:**
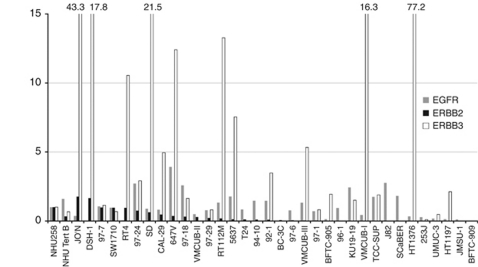
Expression levels of EGFR, ERBB2 and ERBB3 in bladder cell lines. Results represent densitometric measurements of western blot signals relative to ku70 loading control.

Autocrine signalling generated through NRG1 expression even in the presence of low-level receptor expression may have the same cellular consequences as high-level receptor expression. We examined levels of EGFR, ERBB2, ERBB3 and ERBB4 in UC cell lines for comparison with NRG1 levels (Figure 3 and Supplementary Figure 3).

High-level expression of EGFR (⩾2-fold, relative to normal urothelial control) was found in five cell lines, moderate expression (0.1–1.99 × control) in 25 and absent or low expression (⩽0.1 × control) in four. Moderate levels of ERBB2 were detected in 16 cell lines and low levels in 18 cell lines. ERBB3 showed strong expression in 14, moderate expression in 9 and low levels in 11 cell lines. Western blots for ERBB4 demonstrated a single strong band at the predicted size for the full-length ERBB4 protein (180 kDa) only in the breast cancer cell line T47D-positive control (Beerli and Hynes, 1996). No expression of ERBB4 was detected in any of the bladder cancer cell lines or in normal bladder samples. Relationships were sought between expression of NRG1*α* and NRG1*β* and expression of EGFR, ERBB2 and ERBB3 in UC cell lines. There was an inverse correlation between both NRG1*α* and NRG1*β* expression and ERBB3 expression (*P*⩽0.01, Spearman's rank correlation test). No other significant relationships were found.

**Figure 4:**
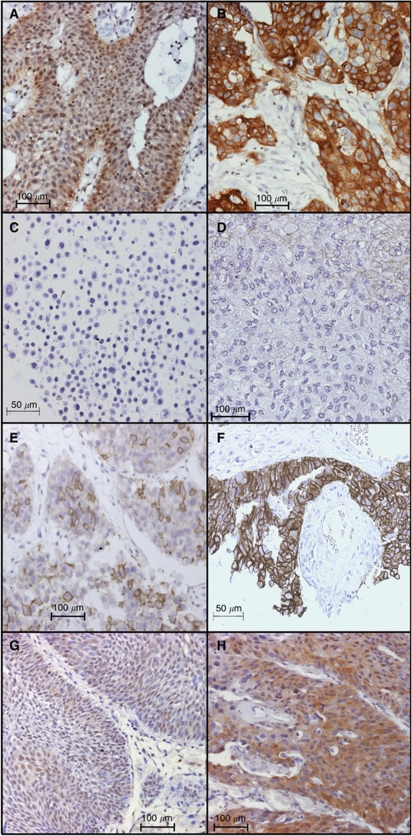
Immunohistochemistry for EGFR and ERBB2 in bladder tumour samples. (**A**) EGFR + (**B**) EGFR +++ (**C**) ERBB2 negative sample (cell pellet from non-expressing cell line); (**D**) ERBB2 + (**E**) ERBB2 ++ (**F**) ERBB2 +++ (**G**) ERBB3 nuclear + and (**H**) ERBB3, cytoplasmic +.

Immunohistochemistry was performed on 54 tumours from the same series as those used for mRNA analysis (Table 1; Figure 4). For one tumour, information on stage was not available. EGFR was detected in the cell membranes of all tumours, 54% of which showed moderate (2+) and 28% showed strong (3+) staining. Increased expression was associated with high grade (Fisher's exact test, *P*=0.05). A marginal association with tumour stage was found when Ta, T1 and ⩾T2 tumours were considered separately (*P*=0.06), and this reached significance (*P*=0.04) when tumours were grouped as non-invasive (Ta) or invasive (T1 or ⩾T2).

ERBB2 membrane staining was detected in 72%, the majority having weak or patchy staining. There was no relationship with grade (*P*=0.12). An association with stage was found (*P*=0.025) but the pattern of this was not clear as results for T1 and ⩾T2 tumours differed and significance was lost when these two groups were combined.

ERBB3 expression was detected in the nucleus, the cytoplasm or both (Figure 4) and was scored separately. There was strong association between tumour grade and cytoplasmic staining (*P*=0.01) but no relationship with nuclear staining (*P*=0.58). There was no association between stage and cytoplasmic or nuclear staining, although there was a trend for more frequent cytoplasmic staining and less frequent nuclear staining with increasing stage. There was no association between cytoplasmic staining or nuclear staining and tumour invasion (*P*=0.40 and 0.41, respectively). Overall, there was an association between nuclear staining and cytoplasmic staining (*P*=0.02) with overall concordance for staining (either both positive or both negative as compared with discordant staining). This pattern could be seen when non-invasive (Ta) and invasive (T1 or ⩾T2) tumours were analysed separately but in neither case did the association reach significance because of limited sample sizes. Logistic regression analysis of the presence of cytoplasmic staining or nuclear staining showed that there was still evidence for concordance (*P*=0.01) after adjustment for invasion. We also examined the relationship of the four possible patterns of ERBB3 staining with grade and stage. There was no relationship with tumour stage but a significant association with grade (*P*=0.01; Supplementary Table 3) was observed. There was evidence of an association between cytoplasmic ERBB3 and ERBB2 positivity (*P*=0.04), but not between nuclear ERBB3 and ERBB2 or between any other ERBB receptor pairs.

Anti-ERBB4 antibodies showed poor specificity on cell pellets with known ERBB4 expression status. Newly constructed cell pellets and different normal tissue samples gave the same results. Therefore, staining was not carried out on tumour sections.

The relationship of NRG1*α* and NRG1*β* mRNA levels to expression of EGFR, ERBB2 and ERBB3 (cytoplasmic or nuclear staining) was examined. No statistically significant associations were found (Spearman's rank correlation test).

## Discussion

ERRB receptor signalling is diverse and flexible in output because of the presence of multiple receptors that homo- or hetero-dimerise and the existence of multiple ligands. Thus, there is much scope for subversion to provide selective advantage in cancer cells. Targeting of overexpressed ERBB2 with the humanised monoclonal antibody Trastuzumab has proven successful in some cases. However, in light of the flexibility of this signalling system, it is not surprising that ERBB2 status alone is not sufficient to predict response. Similarly, expression level of EGFR does not predict response to EGFR inhibitors, although the presence of mutations in the receptor shows correlation with response (Lynch *et al*, 2004; Rosell *et al*, 2006). It is not yet clear whether mutations in ERBB2 (Stephens *et al*, 2004) influence response to ERBB2 inhibitors. Increasingly, it is becoming apparent that the overall molecular context in which such mutations are found significantly influences response to targeted therapies, with molecular lesions downstream of EGFR or ERBB2 indicating likely resistance (Nagata *et al*, 2004; Allegra *et al*, 2009). Similarly, the status of interacting proteins including different members of the ERBB family and their ligands may profoundly influence response.

Prompted by the finding of common genomic alterations in the region of the *NRG1* gene on 8p12 in aggressive bladder cancers and other solid malignancies, as well as the known overexpression of EGFR and ERBB2 in some bladder tumours, we examined the expression of *NRG1* mRNA and protein expression of EGFR, ERBB2, ERBB3 and ERBB4 in bladder tumour tissues and cell lines. There was considerable diversity in the expression of NRG1 in both cell lines and in tumours with higher expression of *NRG1β* than *NRG1α*, suggesting, as proposed previously, that the *β*-isoform may be more biologically important (Lu *et al*, 1995). Overall, tumour tissues showed lower levels of expression than cell lines. Although there was a trend towards upregulation of *NRG1β* in relation to grade and stage, there was no clear relationship with expression of NRG1*β* because of the finding of relatively high levels of expression of NRG1*β* in some pTaG2 tumours (Figure 2). As all of the cell lines apart from RT4 were derived from invasive tumours, and NRG1*β* was high in these, this suggests that increased expression is related to tumour stage. This may become clearer when a larger tumour panel is studied. The possibility that pTaG2 tumours that overexpress NRG1*β* may represent a distinct subset must also be addressed.

Others have found loss of expression and hypermethylation of the NRG1 promoter in breast cancer cell lines that, similar to bladder, commonly show 8p alterations (Chua *et al*, 2009). We found no relationship of expression levels to known genomic alterations (Williams *et al*, 2010) in the cell lines examined here. Although this suggests that copy number alterations and DNA breakpoints close to NRG1 do not affect expression, this does not preclude the possibility that in some cases downregulated expression (for example, tumours 1–9, Figure 1) may be caused by promoter hypermethylation.

A previous study detected NRG1*α* and NRG1*β* mRNA in 90% and 61% of samples, respectively, rates that are remarkably similar to those found here (83 and 66%, respectively; Figure 2) and, as in the present study, levels of NRG1*β* were higher than those of NRG1*α* (Memon *et al*, 2004). One difference is that the primers used to amplify NRG1*β* by Memon *et al* (2004) are predicted to amplify types I and II but not III and the primers we used amplify all three NRG1 types. The similarity in results obtained with these two primer sets suggests that type III neuregulins, which are predicted to mediate juxtacrine rather than paracrine signalling (Falls, 2003), are unlikely to have a significant role in the urothelium.

We found significant coexpression of NRG1*α* and NRG1*β*. The transcriptional regulation of NRG1 is not yet understood but it is possible that these transcripts share the same 5′ sequence and are initiated from the same promoter. Further analysis of the type-specific region, Ig-like regions and spacer sequences that lie upstream of the EGF domain are now required.

There are approximately 20 commercially available antibodies that claim either to be specific to certain isoforms, or to detect all types of NRG1 by means of epitopes in the EGF domain. We attempted to measure NRG1 expression using western blotting with three different NRG1 antibodies, but results were poor, with multiple bands observed that were thought to be non-specific and showed no obvious relationship to mRNA data (data not shown).

Several large studies of EGFR expression using immunohistochemistry are concordant and report detectable levels in 72, 85, 96 and 84% of bladder tumour tissues (Messing, 1990; Wright *et al*, 1991; Lipponen and Eskelinen, 1994; Chow *et al*, 2001) and moderate or strong EGFR expression has been described in 27, 52, 42 and 47% of tumours (Lipponen and Eskelinen, 1994; Mellon *et al*, 1995; Chow *et al*, 2001; Rotterud *et al*, 2005). We detected expression in all samples and this was moderate or strong in 28%.

It is difficult to compare studies with regard to levels of expression as different cut-offs and definitions for ‘expression’ and ‘overexpression’ have been used. The scoring method used here for both EGFR and ERBB2 scoring was chosen because it is extensively and successfully used for ERBB2 in breast cancers in the United Kingdom. We found significantly more intense membrane staining for EGFR in tumours of high grade and stage. Previous studies have reported increased expression in tumours of high grade (Messing, 1990), increased stage (Chow *et al*, 1997) or high grade and stage (Wright *et al*, 1991; Mellon *et al*, 1995) but others have not (Rajkumar *et al*, 1996; Rajjayabun *et al*, 2005).

We found ERBB2 membrane staining in the superficial cells of the normal bladder and ureter and in 72% of tumour tissues. Although moderate (2+) staining was common, only three cases had strong (3+) expression. Similarly, although protein was detected in 47% of cell lines, none had high-level expression. Data on ERBB2 immunohistochemistry in bladder cancer are plentiful and a variety of antibodies, methods and scoring techniques have been used. One recent study applied a standardised methodology used in breast tumour diagnostic assessment to measure both gene amplification and expression of ERBB2 (Lae *et al*, 2010). Only a few tumours (5%) expressed levels as high as those that are used to select breast cancers for Trastuzumab treatment (3+). Our finding of 5.5% is compatible with this. We found no significant relationship between ERBB2 expression and tumour grade but an association with tumour invasion was apparent.

We found detectable ERRB3 by western blotting in normal urothelial cells and in 68% of tumour cell lines, with high levels in 41%. Immunohistochemistry of tumour tissues has previously found moderate or strong ERBB3 expression in 11–49% (Rajkumar *et al*, 1996; Chow *et al*, 1997, 2001; Rotterud *et al*, 2005). Here we scored cytoplasmic and nuclear staining separately and found that 37 and 48% of tumours were positive, respectively. Cytoplasmic staining was associated with tumour grade as observed previously (Chow *et al*, 2001). There was also an association with ERBB2 positivity (*P*=0.04). Previously, a relationship of ERBB2 and ERBB3 positivity has been reported to be associated with poor patient survival and may indicate a role for signalling by ERBB2/3 heterodimers (Chow *et al*, 2001).

The significance of nuclear *vs* cytoplasmic localisation of ERBB3 protein is not clear. It has been reported that the protein is nuclear in cultured normal mammary epithelial cells and that stimulation with NRG*β*1 causes shuttling to the cytoplasm (Offterdinger *et al*, 2002). We did not observe nuclear staining in normal urothelium and found no evidence for increased cytoplasmic staining in tumours with higher levels of NRG1 expression. In contrast, in prostate tissues, nuclear ERBB3 is reported only in cancer tissues and not in normal tissues, increasing with Gleason grade and disease recurrence (Koumakpayi *et al*, 2006, 2007). Here we found no association of nuclear staining with either grade or stage. A larger study is merited to establish the significance of these different staining patterns in bladder cancer.

NRG1 expression was inversely related to ERBB3 expression in UC cell lines. This could indicate that expression of either the ligand or the receptor may be important. Indeed, of the seven cell lines with greatest total NRG1 expression shown in Figure 1, all but one (SW1710) show very low or undetectable levels of either ERBB2 or ERBB3. This may identify the presence of an NRG1–ERBB3/ERBB2 autocrine loop in these cases as in other tumour cell types (Gilmour *et al*, 2002; Venkateswarlu *et al*, 2002; Li *et al*, 2004; Sheng *et al*, 2010), and could indicate that very low levels of receptor expression may be sufficient for signalling. This relationship was not seen in tumours, although it should be noted that the UC cell lines used were derived from invasive tumours and may represent a highly selected subgroup. Therefore, analysis of a much larger group of invasive tumours is merited.

Already, it is clear that expression of NRG can influence tumour response to Herceptin. Knowledge of ligand expression status will also be critically important in assessing likely response to agents such as Pertuzumab that interfere with ligand-dependent signalling through ERBB2 (Agus *et al*, 2002). Our finding that NRG1 expression is higher in tumours of high grade and stage may be particularly relevant in this context, as these are tumours for which targeted therapies are most urgently needed. In conclusion, our results suggest that NRG1–ERBB receptor interactions may have a significant role in the pathogenesis of bladder cancer and are potential targets for therapy. The identification of tumour cell lines with different patterns of expression of these proteins now provides the opportunity to carry out functional analyses to test this.

## Figures and Tables

**Table 1:** Expression of EGFR, ERBB2 and ERBB3 in bladder tumours according to stage and grade

	**EGFR**			**ERBB2**				**ERBB3 cytoplasmic**		**ERBB3 nuclear**	
**Stage/grade**	**1+ (%)**	**2+ (%)**	**3+ (%)**	**− (%)**	**1+ (%)**	**2+ (%)**	**3+ (%)**	**− (%)**	**+ (%)**	**0 (%)**	**+ (%)**
G2	8 (33.33)	11(45.83)	5 (20.53)	9 (37.5)	13 (54.17)	2 (8.33)	0	20 (83.33)	4 (16.67)	11 (45.83)	13 (54.17)
G3	2 (6.67)	18 (60.0)	10 (33.33)^a^	6 (20.0)	14 (46.67)	8 (26.67)	2 (6.67)	14 (46.67)	16 (53.33)^b^	17 (56.67)	13 (43.33)
Ta	8 (34.78)	10 (43.48)	5 (21.74)	9 (39.13)	11 (47.83)	3 (13.04)	0	16 (69.57)	7 (30.43)	10 (43.48)	13 (56.52)
T1	2 (11.11)	12 (66.67)	4 (22.22)	1 (5.56)	11 (61.11)	6 (33.33)	0	11 (61.11)	7 (38.89)	10 (55.56)	8 (44.44)
⩾T2	0	6 (50.0)	6 (50.0)^c^	4 (33.33)	5 (41.67)	1 (8.33)	2 (6.67)^d^	6 (50.0)	6 (50.0)	7 (58.33)	5 (41.57)

a *P*=0.05.

b *P*=0.01.

c When invasive tumours (T1+⩾T2) grouped *P*=0.04.

d *P*=0.025 when Ta, T1 and ⩾T2 analysed separately; *P*=0.225 when invasive tumours (T1+⩾T2) grouped.
