# Supplementary material for: Expression of NRG1 and its receptors in human bladder cancer
Source: Br J Cancer. 2011 Mar 1;104(7):1135–43. doi: 10.1038/bjc.2011.39 (PMC3068491; doi:10.1038/bjc.2011.39)
Supplement: Supplementary Figure 1 [file bjc201139x1.pdf]

Supplementary Figure 1. NRG1 patterns of alternative splicing

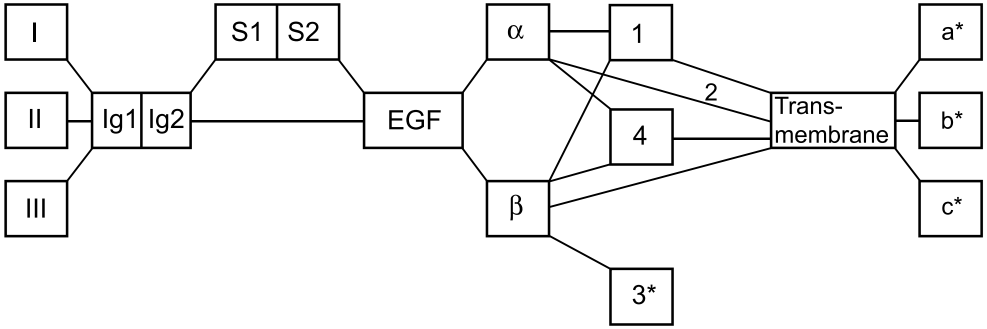

S1 and S2, "spacer"; EGF α and β, EGF domain; 1, 3 and 4, "stalk"; *, stop codon.
