# Supplementary material for: Expression of NRG1 and its receptors in human bladder cancer
Source: Br J Cancer. 2011 Mar 1;104(7):1135–43. doi: 10.1038/bjc.2011.39 (PMC3068491; doi:10.1038/bjc.2011.39)
Supplement: Supplementary Figure 2 [file bjc201139x2.pdf]

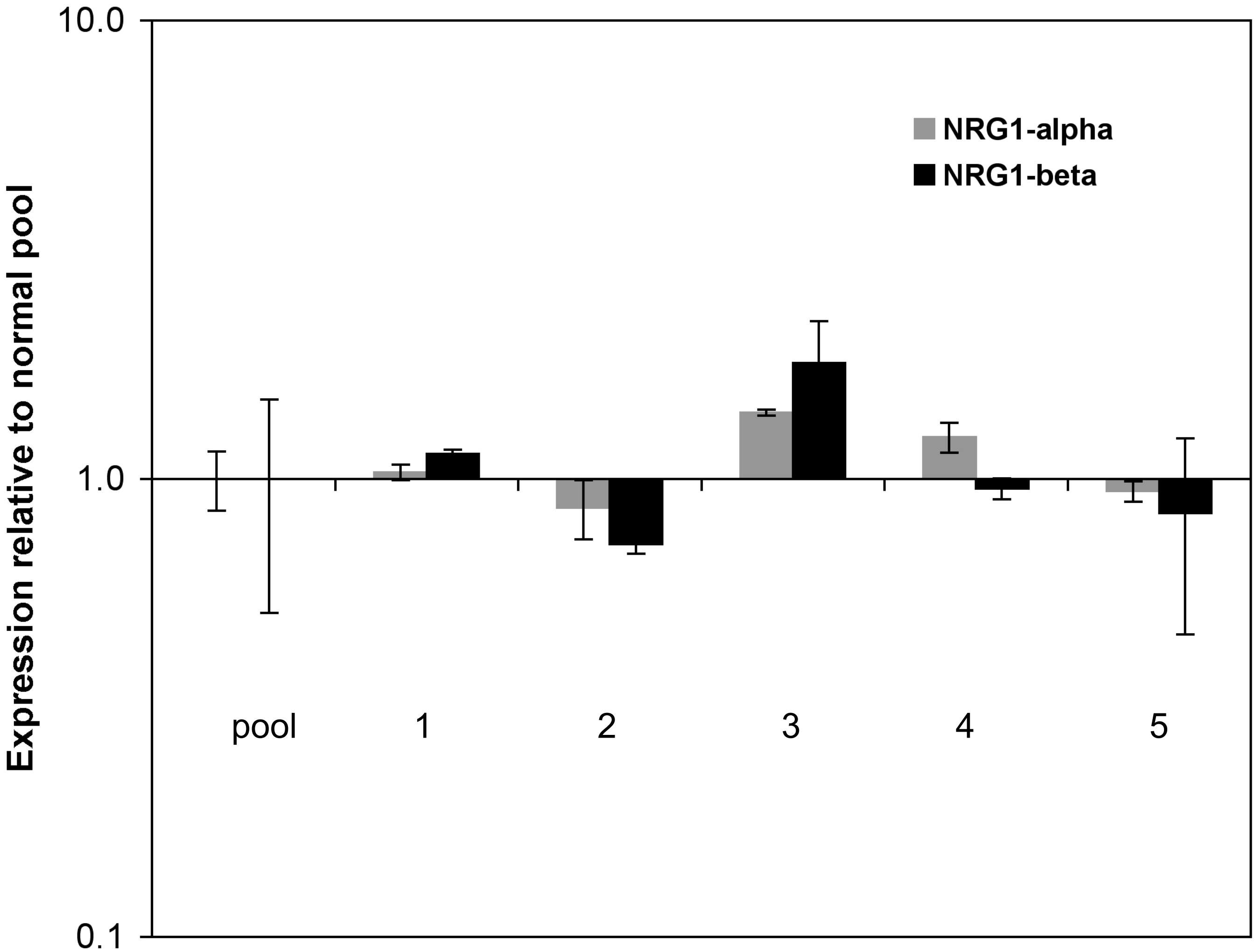

Supplementary Figure 2. Real-time RT-PCR measurements of NRGα and NRGβ levels in samples of normal urothelial cells isolated from normal human ureter.
Five individual samples were compared to pooled RNA from the same samples.
