# Supplementary material for: Expression of NRG1 and its receptors in human bladder cancer
Source: Br J Cancer. 2011 Mar 1;104(7):1135–43. doi: 10.1038/bjc.2011.39 (PMC3068491; doi:10.1038/bjc.2011.39)
Supplement: Supplementary Figure 3 [file bjc201139x3.pdf]

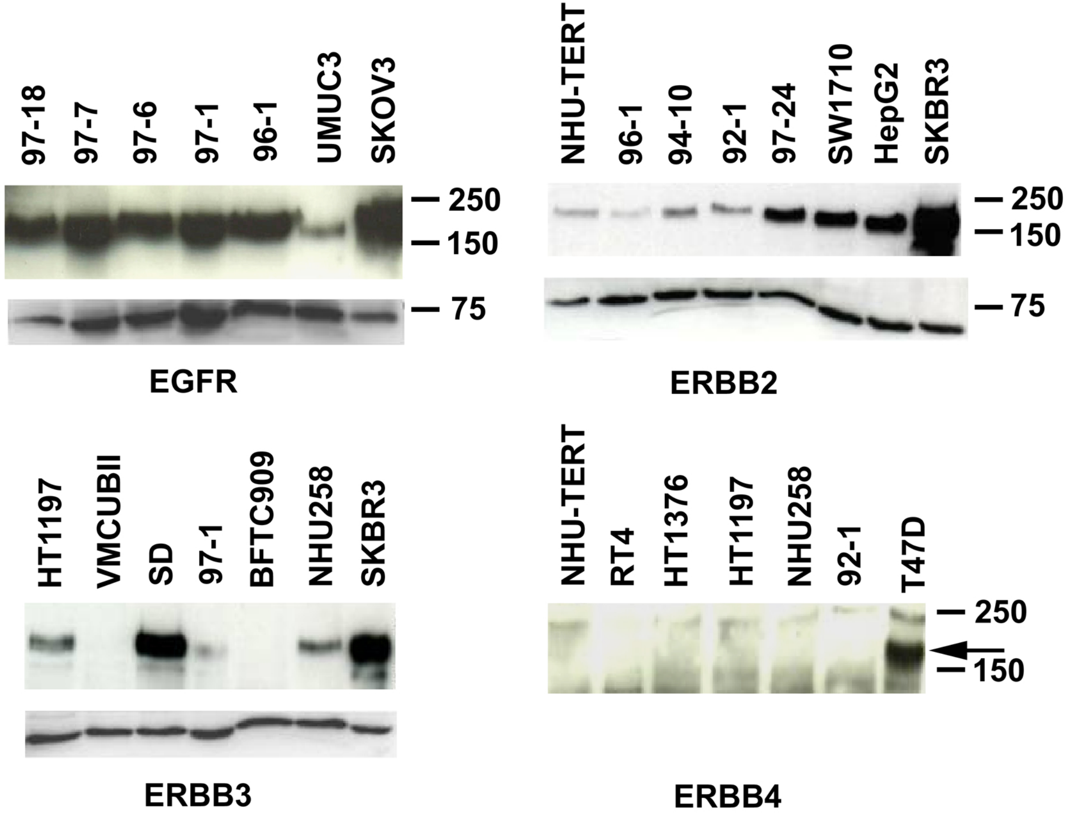

Supplementary Figure 3. Western blots showing ERBB protein expression in cell lines
Lower panels, ku70 loading control.
SKOV3, SKBR3 and T47D are non-urothelial cell lines with known high expression of ERBB receptors, used as positive controls.
