# Supplementary material for: Expression of NRG1 and its receptors in human bladder cancer
Source: Br J Cancer. 2011 Mar 1;104(7):1135–43. doi: 10.1038/bjc.2011.39 (PMC3068491; doi:10.1038/bjc.2011.39)
Supplement: Supplementary Table 1 [file bjc201139x4.doc]

**Supplementary Table 1. Cell lines and their origins.**

| Cell line | Grade/  stage | Other information |
| --- | --- | --- |
| TERT-NHUC B |  | Immortalised normal urothelial cell strain B |
| NHU258 |  | Normal urothelium primary mortal cell strain |
| T24 | G3 | Primary tumour, bladder |
| J82 | G3 | Primary tumour, bladder |
| 5637 |  | Primary tumour, bladder |
| 647V | G2 | Primary tumour, bladder |
| VM-CUB-I |  | Primary tumour, bladder |
| VM-CUB-II |  | Metastatic lymph node |
| VM-CUB-III |  | Primary tumour, bladder |
| 253J | G4 T4 | Metastatic lymph node |
| RT4 cl5 | G1 T2 | Recurrence, bladder |
| RT112M | G2 | Primary tumour, bladder |
| HT1197 |  | Bladder |
| HT1376 |  | Bladder |
| SD |  | Primary tumour, bladder |
| UMUC3 |  | Bladder |
| SW1710 | G3 | Bladder |
| BFTC905 | G3 | Primary tumour, bladder |
| BFTC909 | G3 | Bladder |
| JO’N |  | Bladder |
| DSH1 | T1a G2 | Recurrence, bladder |
| JMSU-1 |  | Bladder |
| CAL-29 |  | Bladder |
| SCaBER | SCC | Bladder SCC |
| TCC-SUP | G4 | Bladder |
| BC-3C | G4 | Bladder |
| KU19-19 | G3 T3b | Bladder |
| 92-1 | T4 G3 | Bladder |
| 94-10 | T3 G2-3 | Bladder |
| 96-1 | T3 G2-3 | Bladder |
| 97-1 | T1/2 G1 | Bladder |
| 97-6 | T3 G3 | Bladder |
| 97-7 | T1 G2-3 | Bladder |
| 97-18 | T2 G3 | Bladder |
| 97-24 | T3 G3 | Bladder |
| 97-29 | T1 G1-2 | Bladder |

^1^References to initial descriptions of cell lines are given in (Catt*an et* al, 2001; Chapm*an et* al, 2006; Ellio*tt et* al, 1974; Mori*ta et* al, 1995; Nay*ak et* al, 1977; O'Too*le et* al, 1976; Rashe*ed et* al, 1977; Sark*ar et* al, 2000; Tachiba*na et* al, 1995; Tze*ng et* al, 1996; Williams, 1980; Willia*ms et* al, 2002; Yeag*er et* al, 1998)
