# Supplementary material for: Expression of NRG1 and its receptors in human bladder cancer
Source: Br J Cancer. 2011 Mar 1;104(7):1135–43. doi: 10.1038/bjc.2011.39 (PMC3068491; doi:10.1038/bjc.2011.39)
Supplement: Supplementary Table 2 [file bjc201139x5.doc]

**Supplementary Table 2. Primers used for qRT-PCR.**

| **Primer** | **Forward Sequence (5’-3’)** | **Reverse Sequence (5’-3’)** |
| --- | --- | --- |
| *NRG1* alpha | ACCTTTCAAACCCCTCGAGATAC | TCATGGGCACATTCTCAGTACAT |
| *NRG1* beta | GCTTCATGGTGAAAGACCTTTCA | ATTACGTAGTTTTGGCAGCGATC |
| SDHA | TGGGAACAAGAGGGCATCTG | CCACCACTGCATCAAATTCATG |
| HPRT1 | GACACTGGCAAAACAATGCA | CTTCGTGGGGTCCTTTTCACC |
