# Supplementary material for: Expression of NRG1 and its receptors in human bladder cancer
Source: Br J Cancer. 2011 Mar 1;104(7):1135–43. doi: 10.1038/bjc.2011.39 (PMC3068491; doi:10.1038/bjc.2011.39)
Supplement: Supplementary Table 3 [file bjc201139x6.doc]

**Supplementary Table 3. Relationship of ERBB3 expression patterns to tumour grade.**

|  | **Grade** | |  |
| --- | --- | --- | --- |
| **ERBB3** | **2** | **3** | **Total** |
| ++^1^ | 4 | 10 | 14 |
| +- | 0 | 6 | 6 |
| -+ | 9 | 3 | 12 |
| -- | 11 | 11 | 22 |
| **Total** | **24** | **30** | **54** |

Fisher’s exact p=0.01

^1^ cytoplasmic/nuclear
